# Previous COVID‐19 Vaccination Modulates Type I Interferon and Natural Killer Cell Responses During SARS‐CoV‐2 Infection

**DOI:** 10.1111/jcmm.71190

**Published:** 2026-05-22

**Authors:** Luca Maddaloni, Valentina Tirelli, Ginevra Bugani, Letizia Santinelli, Matteo Fracella, Mario Picozza, Eugenio N. Cavallari, Giancarlo Ceccarelli, Guido Antonelli, Claudio M. Mastroianni, Carolina Scagnolari, Gabriella d'Ettorre

**Affiliations:** ^1^ Department of Molecular Medicine Sapienza University of Rome Rome Italy; ^2^ Department of Public Health and Infectious Diseases Sapienza University of Rome Rome Italy; ^3^ Core Facilities Istituto Superiore Di Sanità Rome Italy; ^4^ Department of Molecular Medicine, Laboratory of Virology Sapienza University of Rome Rome Italy; ^5^ Azienda Ospedaliero Universitaria Policlinico Umberto I Rome Italy; ^6^ Department of Molecular Medicine, Microbiology and Virology Laboratory Sapienza University of Rome Rome Italy; ^7^ Istituto Pasteur Italia‐Fondazione Cenci Bolognetti Rome Italy

**Keywords:** COVID‐19 vaccine, IFN‐I, innate immunity, NK cells, SARS‐CoV‐2

## Abstract

The impact of the SARS‐CoV‐2 vaccine on innate immunity is not well understood. However, it has played a pivotal role in reducing COVID‐19 severity and mortality. Recent findings have revealed that vaccine efficacy is influenced not only by the effective activation of adaptive immunity, but also by the modulation of innate immunity. This cross‐sectional study evaluates the natural killer (NK) cell response and its relationship with type I interferon (IFN‐I) gene expression in SARS‐CoV‐2‐infected patients who had previously received the anti‐spike vaccine, as well as in unvaccinated patients. Vaccinated individuals showed a higher frequency of NK CD56^dim^CD16^−^ cells and increased IFN‐α2 and IFN‐ω mRNA expression (*p* < 0.05). By contrast, unvaccinated patients displayed a predominance of NK CD56^dim^CD16^+^ cells and reduced IFN‐I gene expression (*p* < 0.05). A positive correlation was found between IFN‐I levels and the frequency of NK CD56^dim^CD16^−^ cells and a negative correlation between IFN‐I levels and NK CD56^dim^CD16^+^ cells. Furthermore, despite having more comorbidities, vaccinated patients had faster SARS‐CoV‐2 clearance, which reinforces the immunological advantage conferred by vaccination. Together, these findings suggest that the SARS‐CoV‐2 vaccine can modify the innate immune response by enhancing the NK cell response and increasing the magnitude of IFN‐I production during SARS‐CoV‐2 infection.

## 1 Introduction

In the context of the COVID‐19 pandemic, the production and distribution of vaccines were critical in limiting the rate and severity of SARS‐CoV‐2 infection, as well as hospitalization and mortality [1, 2]. Anti‐SARS‐CoV‐2 vaccination can induce both antibody production and specific T‐cell responses [3], but an intriguing novel aspect is its ability to modulate components of innate immunity, such as natural killer (NK) cells [4]. Some recent studies have shown that NK cells are activated by anti‐spike (S) vaccination or SARS‐CoV‐2 natural infection [5, 6], and that they are more active after re‐infection [7]. In addition, NK cell‐mediated antibody‐dependent cellular cytotoxicity (ADCC) activity, which plays a key role in COVID‐19, has been reported to be triggered by antibodies induced by natural SARS‐CoV‐2 infection or anti‐S vaccination [8].

Among the components of innate immunity, interferons (IFNs) are able to stimulate NK cell activation and significantly suppress SARS‐CoV‐2 replication [9, 10, 11, 12]. Data from the literature and from our previous studies have shown that severe COVID‐19 can occur with either low and excessive type I/III IFN and IFN‐stimulated genes production [13, 14, 15, 16]. It is noteworthy that severe forms of COVID‐19 have been associated with dysregulation of IFN due to viral evasion mechanisms, temporal defects in IFN production, host genetic factors or the development of anti‐IFN neutralizing autoantibodies [17, 18, 19, 20, 21]. Recent data also suggest that these cytokines could be important during SARS‐CoV‐2 vaccination, with a positive correlation observed between the IFN gene signature and the level of anti‐S antibody production following vaccination [22]. We have previously observed that COVID‐19 patients who had been vaccinated against SARS‐CoV‐2 exhibit higher IFN‐I mRNA levels than unvaccinated patients and show more effective and faster resolution of infection [23].

Given the emerging novel immunological effects of anti‐S vaccination [3], and the role of NK cells in antibody‐mediated SARS‐CoV‐2 clearance [8], we investigated how prior anti‐S vaccination might affect the NK cell response during SARS‐CoV‐2 infection. Therefore, we aimed to evaluate the NK cell response in SARS‐CoV‐2 patients previously vaccinated with the anti‐S vaccine and in unvaccinated patients. Specifically, NK cell subsets (CD56^dim^CD16^+^, CD56^dim^CD16^−^, CD56^bright^CD16^+^ and CD56^bright^CD16^−^) were investigated in peripheral blood mononuclear cells (PBMCs) from SARS‐CoV‐2 infected patients. To gain further insight into the role of IFN changes during anti‐S vaccination, we also extended our previous study [23], by investigating the relationship between NK cell response and IFN‐α2 and IFN‐ω mRNA levels.

## 2 Materials and Methods

### 2.1 Participants

This cross‐sectional study was conducted at the Division of Infectious Diseases, Department of Public Health and Infectious Diseases, Umberto I Hospital of the Sapienza University of Rome (Italy), and included 69 outpatients with PCR test confirmed SARS‐CoV‐2 infection from nasopharyngeal swabs from 21 April to 10 December 2021. Patients informations were obtained from medical records in the hospital's electronic information system. All patients were high risk individuals treated with monoclonal antibodies in a single administration (casirivimab 1200 mg + imdevimab 1200 mg or bamlanivimab 700 mg + etesevimab 1400 mg). The following variables were included in the study: age, sex, vaccination status against SARS‐CoV‐2 and Charlson Comorbidity Index [24]. The study was performed following the Institutional Review Board (Department of Public Health and Infectious Diseases, Sapienza, University of Rome) and the Ethics Committee (Comitato Etico Territoriale Lazio Area 1) approval and the signing of informed consent by all study participants.

### 2.2 Blood Samples Collection

Fresh peripheral blood samples were collected by venipuncture into Vacutainer tubes containing a gel separator for serum or EDTA (BD Biosciences, USA) from SARS‐CoV‐2‐infected patients at least 48 h after diagnosis and before monoclonal antibody treatment. The first were centrifuged at 3000 rpm for 10 min, the serum was collected and stored at **−**80°C. The latter were centrifuged at 1500 rpm for 10 min to isolate plasma (stored at **−**80°C) and further processed by Ficoll gradient centrifugation (Lympholyte; Cedarlane Labs, Canada) to obtain PBMCs. PBMCs were washed twice in phosphate‐buffered saline (PBS) and stored in foetal bovine serum (FBS) supplemented with 10% dimethyl sulfoxide (DMSO) in liquid nitrogen until use.

### 2.3 SARS‐CoV‐2 Infection Diagnosis and Anti‐Spike Antibody Titre Quantification

SARS‐CoV‐2 infection was diagnosed through extraction of viral RNA from nasopharyngeal swabs (Versant SP 1.0 Kit, Siemens Healthineers, Germany), reverse‐transcription and real‐time PCR amplification of E and S viral genes (RealStar SARS‐CoV‐2 RT PCR, Altona Diagnostics, Germany). Type G immunoglobulins (IgGs) against SARS‐CoV‐2 S protein were determined in serum from infected patients using a commercial assay (LIAISON SARS‐CoV‐2 TrimericS IgG, DiaSorin, Italy). The anti‐S antibody titers provided by the assay are expressed as binding antibody units per ml (BAU/ml), ranging between 4.81 and 2080 BAU/mL. Values < 33.8 BAU/ml are considered negative according to manufacturer's instructions. Specimens containing anti‐TrimericS IgG levels over the assay measuring range (> 2080 BAU/mL) were automatically diluted with a factor of 1:10 using the LIAISON TrimericS IgG Diluent Accessory.

### 2.4 Immunophenotyping by Multiparametric Flow Cytometry Assay

**FIGURE 1:**
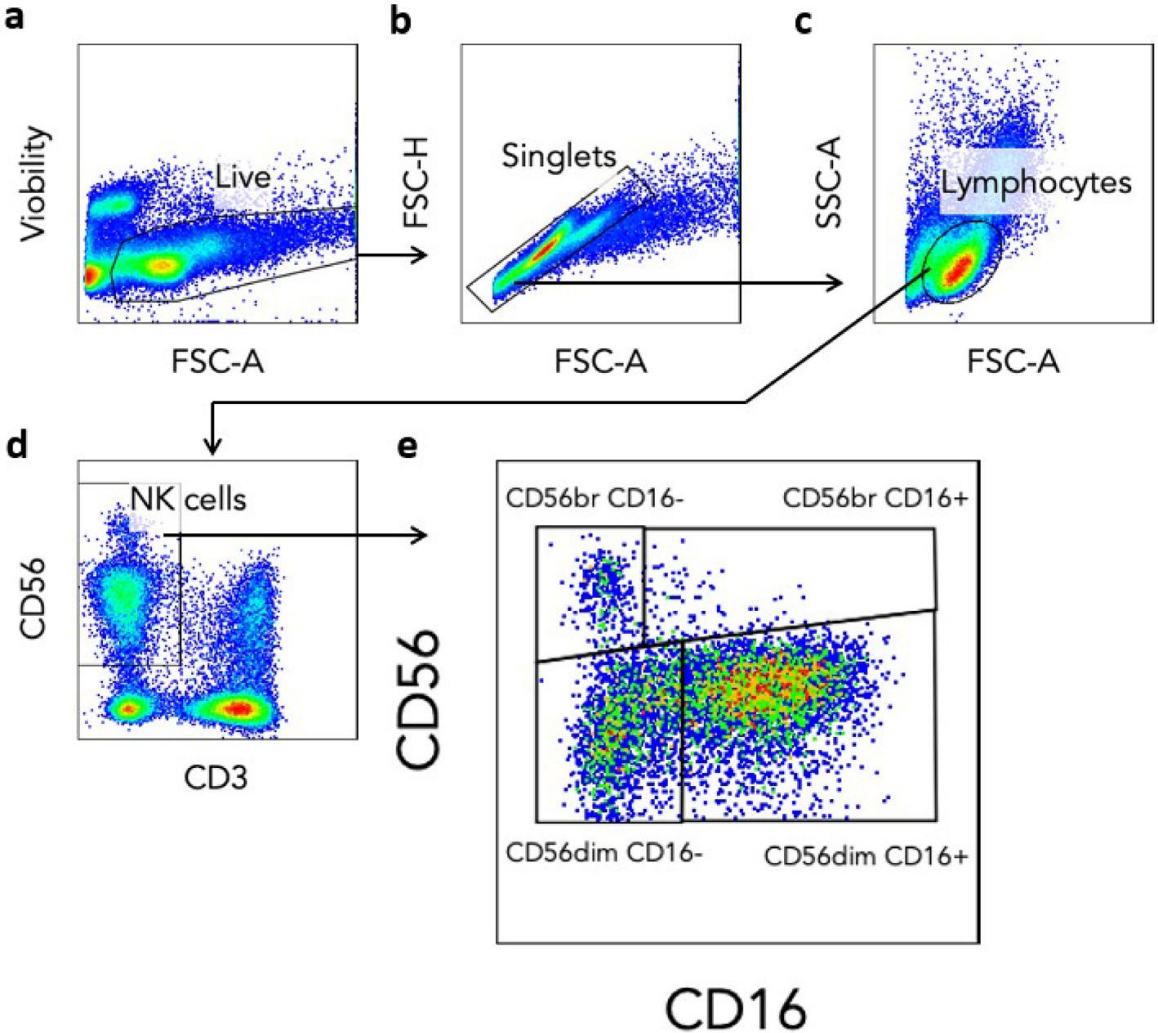
Representative flow cytometry plots showing the gating strategy used to analyse NK cell subsets in PBMC samples. NK cells were selected via sequential gating by the exclusion of dead cells (a) and doublets (b), followed by positive gating on lymphocytes (c) and CD3^−^CD56^+^ cells (d). Then, NK subsets were selected based on differential expression of CD56 and CD16 (e).

Thawed PBMCs were washed twice with PBS and the distribution of cellular phenotypes was evaluated by Miltenyi Biotec flow cytometer‐MACSQuant Analyser (eight fluorescence channels, three lasers; Miltenyi Biotec, Germany). After defining cell viability by excluding dead cells using Viobility 488/520 Fixable Dye (Miltenyi Biotec), cellular phenotypes were obtained employing different fluorochrome‐labelled anti‐human monoclonal antibodies: CD3‐PerCP‐Vio770, CD16‐PE‐Vio770 and CD56‐PE (Miltenyi Biotec). NK cell subsets were identified as CD3^−^CD56^+^ and were further subdivided into CD56^dim^CD16^+^, CD56^dim^CD16^**−**^, CD56^bright^CD16^+^ and CD56^bright^CD16^**−**^. A minimum of 10^5^ events were acquired for each sample. Gating strategies and data analysis were performed using FlowJo v10.10 (Becton Dickinson, USA). Representative flow cytometry plots illustrating the gating strategy are shown in Figure 1.

### 2.5 Bioassay for Detection of Neutralizing Antibodies Against IFN‐I

A neutralization assay was performed to assess the presence of autoantibodies that bind the active site of IFN and block its biological activity [25]. The biological system used in this assay is based on the detection of the cytopathic effect of murine encephalomyocarditis virus (EMCV) on the A549 cell line, as previously described [20]. Neutralizing autoantibodies (nAbs) against IFN‐α2a (Roferon‐A, Roche, Switzerland), IFN‐α2b (Intron, Merck & Co., USA), and IFN‐ω (PBL Assay Science, USA) were measured in the decomplemented sera and expressed as tenfold reduction units (TRU)/mL, as previously reported [25].

### 2.6 Real‐Time PCR for mRNA Expression Analysis

Total RNA was extracted from PBMCs using Direct‐zol RNA Miniprep Kit (Zymo Research, USA) and reverse‐transcribed using the High‐Capacity cDNA Reverse Transcription Kit (Applied Biosystems, USA), according to the manufacturer's protocol. IFN‐α2 and IFN‐ω mRNA expression levels relative to the β‐glucuronidase housekeeping gene were quantified through real‐time PCR using the LightCycler480 Instrument II (Roche), as previously described [23].

### 2.7 Statistical Analysis

Patients' data were presented as median (interquartile range) or number (percentage). Demographic, virological, serological, and clinical characteristics of patients were analysed using the ‘N‐1’ Chi‐squared test. Cross‐sectional data comparing vaccinated and unvaccinated patients were analysed with the Mann–Whitney U test. The Spearman's rho coefficient was calculated to determine the correlation between gene expression levels and cell frequencies. Statistical analyses were performed using GraphPad Prism software, version 9.4 (GraphPad Software Inc., USA) and a *p*‐value of less than 0.05 was considered statistically significant.

## 3 Results

### 3.1 Study Population

A total of 69 SARS‐CoV‐2 infected patients were enrolled in this study and divided into two groups according to anti‐S vaccination status: vaccinated (*n* = 47) and unvaccinated (*n* = 22). The demographic and clinical characteristics of these two groups are shown in Table 1; the type of anti‐SARS‐CoV‐2 vaccine and number of doses previously received are described in Table S1. The median elapsed time since vaccination was 5 months, with an interquartile range between 3 and 7. None of the patients reported previous SARS‐CoV‐2 infection. None of the unvaccinated patients had detectable anti‐S antibodies in their serum, whereas all vaccinated patients tested positive for these antibodies. RT‐PCR SARS‐CoV‐2 RNA test data were available 12 days after enrolment for 40 vaccinated and 18 unvaccinated individuals. The vaccinated group had a higher rate of negative RT‐PCR tests for SARS‐CoV‐2 than the unvaccinated group (32.5% vs. 0%, *p* = 0.0065) after monoclonal antibody therapy (Table 1).

**TABLE 1:** Demographic, virological, serological and clinical characteristics of SARS‐CoV‐2 infected patients.

Parameters	SARS‐CoV‐2 vaccinated patients (47/69, 68%)		SARS‐CoV‐2 unvaccinated patients (22/69, 32%)		*p*
	Median (IQR 25%–75%)	*n* (%)	Median (IQR 25%–75%)	*n* (%)	
Sex assigned at birth					
Male	—	23 (48.9)	—	10 (45.5)	0.758
Female	—	24 (51.1)	—	12 (54.5)	0.758
Age (years)	66 (60–76)	—	57 (53–73)	—	0.071
Charlson Comorbidity Index^a^	3 (3–4)	—	2 (2–3)	—	**0.003**
Anti‐spike (S) antibodies test					
Undetectable (< 33.8 BAU/ml)	—	0 (0)	—	22 (100)	**< 0.0001**
Detectable (> 33.8 BAU/ml)	—	47 (100)	—	0 (0)	**< 0.0001**
Follow‐up RT‐PCR SARS‐CoV‐2‐test^b^					
Positive	—	27 (67.5)	—	18 (100)	**0.0065**
Negative	—	13 (32.5)	—	0 (0)	**0.0065**

*Note:* Data are expressed as median (interquartile range) or number (percentage) and were analysed using Mann–Whitney U test and ‘N‐1’ chi‐squared test, respectively. *p‐*values < 0.05 were considered statistically significant and are shown in bold.

^a^ The Charlson Comorbidity Index predicts the mortality for a patient who may have a number of comorbidities [24].

^b^ Data were available for 40 vaccinated and 18 unvaccinated patients, respectively, 12 days following a single administration of monoclonal antibody therapy.

### 3.2 Frequencies of NK Cell Subsets in SARS‐CoV‐2 Infected Patients According to the Vaccination Status

**FIGURE 2:**
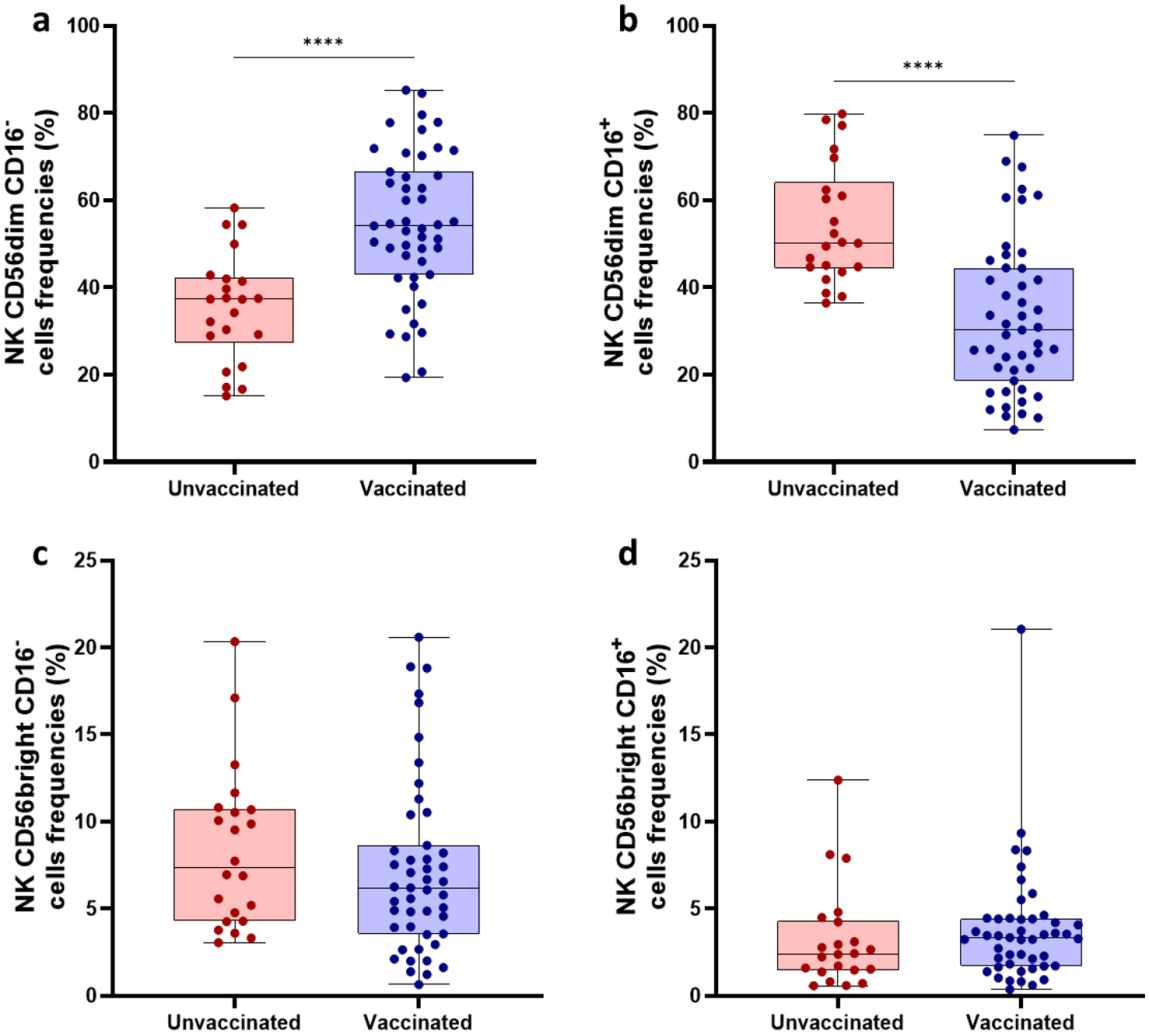
Comparison of NK CD56^dim^CD16^**−**^ (a), NK CD56^dim^CD16^+^ (b), NK CD56^bright^CD16^**−**^ (c) and NK CD56^bright^CD16^+^ (d) cells frequencies between vaccinated and unvaccinated SARS‐CoV‐2 infected patients. Data were analysed using the Mann‐Whitney U test. ****p < 0.0001.

The effect of previous SARS‐CoV‐2 vaccination on the distribution of NK cell subsets was investigated by stratifying all SARS‐CoV‐2 infected patients according to their vaccination status. Unvaccinated patients had a lower percentage of NK CD56^dim^CD16^−^ cells compared to vaccinated patients (*p* < 0.0001), but an increased frequency of NK CD56^dim^CD16^+^ cells (*p* < 0.0001) (Figure 2a,b). The percentage of NK CD56^bright^CD16^+/−^ cells was similar between the two groups of SARS‐CoV‐2 positive patients (Figure 2c,d).

### 3.3 Correlation Analysis Between NK Cells Frequencies and IFN‐I Expression

**FIGURE 3:**
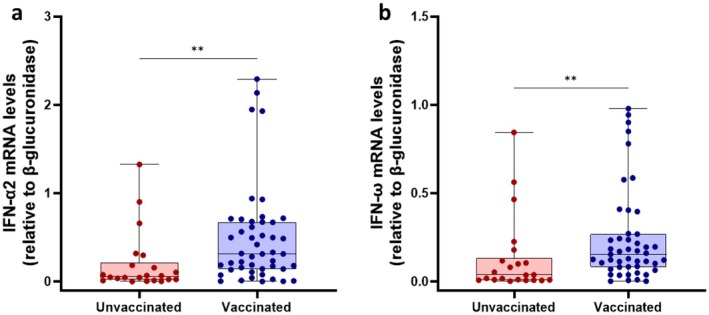
Comparison of IFN‐α2 (a) and IFN‐ω (b) mRNA expression levels between vaccinated and unvaccinated SARS‐CoV‐2 infected patients. Data were analysed using the Mann–Whitney U test. **p < 0.01.

**FIGURE 4:**
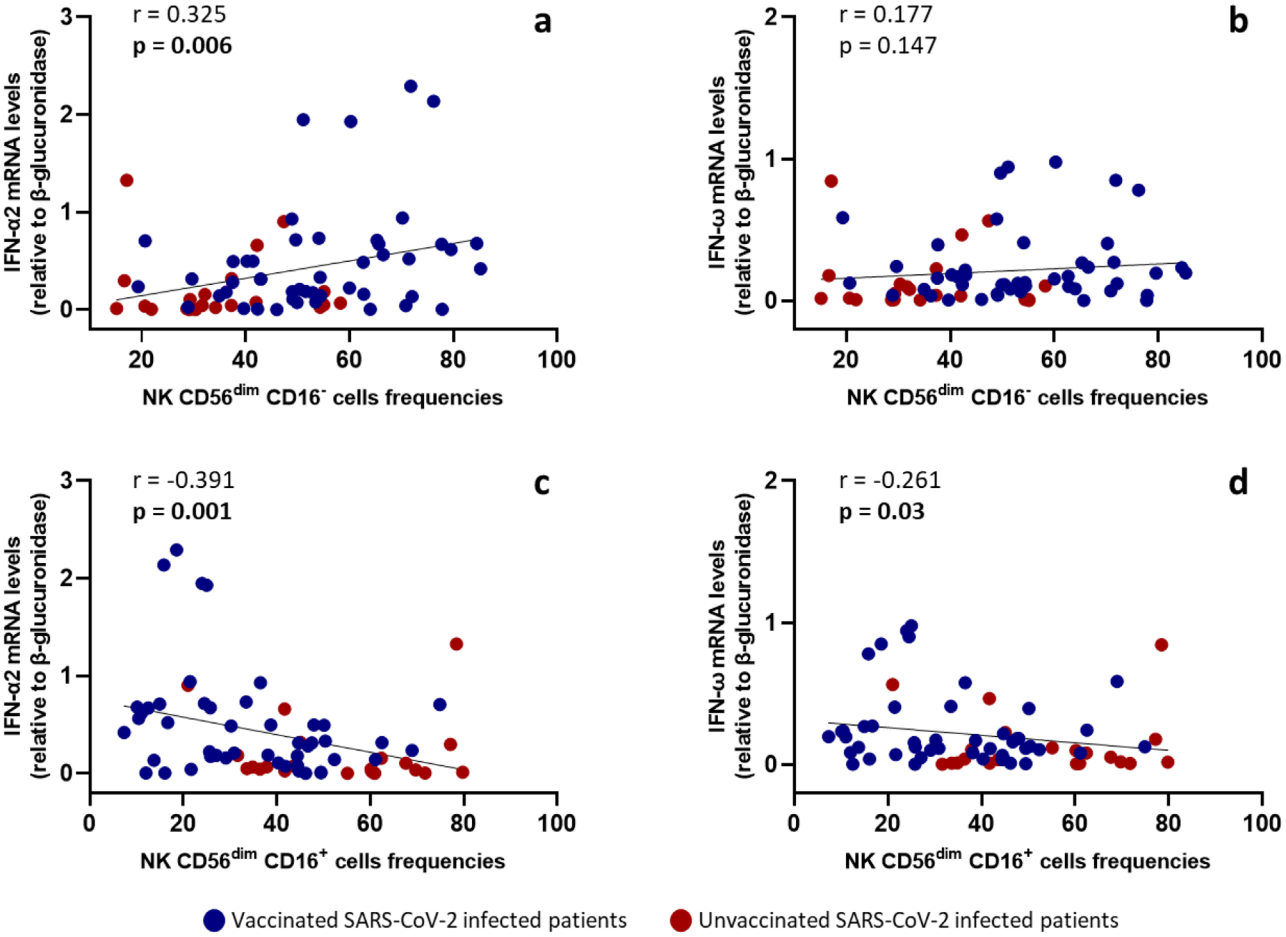
Correlation between NK CD56^dim^ cells frequencies and IFN‐I mRNA expression levels. Data were analysed using Spearman's rank correlation test and *p* < 0.05 was considered statistically significant.

Considering the strong association between IFN‐I and NK cell activity [9, 10] and given the observed differences in NK CD56^dim^ cells between vaccinated and unvaccinated patients, a correlation analysis was performed between IFN‐I mRNA expression levels and NK cell subsets frequencies. Specifically, IFN‐α2 and IFN‐ω mRNA expression data were obtained from our previous study [23]. To avoid confounding factors that could affect this analysis, we tested for the presence of anti‐IFN‐I nAbs in serum samples from these participants. All serum samples were negative for anti‐IFN‐α2a/b and IFN‐ω (< 10 TRU/mL). Results confirmed our previous analysis that the unvaccinated group showed reduced IFN‐α2 (*p* = 0.0015) and IFN‐ω (*p* = 0.003) mRNA expression levels in PBMCs compared to the vaccinated group (Figure 3a,b). A positive correlation was found between NK CD56^dim^CD16^**−**^ cells and IFN‐α2 (*r* = 0.325; *p* = 0.006), whereas NK CD56^dim^CD16^+^ cells showed a negative correlation with IFN‐α2 (*r* = −0.391; *p* = 0.001) and IFN‐ω (*r* = ^**−**^0.261; *p* = 0.03). Correlation coefficients (*r*) and *p*‐values (*p*) are shown in Figure 4a–d.

## 4 Discussion

In the context of the COVID‐19 pandemic, vaccination against SARS‐CoV‐2 was critical in reducing severe clinical outcomes by promoting an effective and long‐lasting immune response. While the stimulation of both T‐ and B‐lymphocytes by the SARS‐CoV‐2 vaccine has been well described [3], considerable interest has arisen in understanding the role of innate immunity. The ability of the innate immune system to develop a memory‐like response after exposure to certain pathogens or vaccines has recently been described for several microorganisms. This phenomenon is defined as ‘trained innate immunity’ [26]. Therefore, the first focus of our study was the evaluation of NK cell response in SARS‐CoV‐2‐infected patients who had or had not previously been vaccinated against the S protein. Vaccinated patients had a higher Charlson Comorbidity Index than unvaccinated patients, reflecting a greater risk of developing severe symptoms of the disease. These data reflect the vaccination strategy adopted during the pandemic, which prioritized individuals at greatest risk of severe COVID‐19.

Vaccinated patients were characterized by an increased percentage of NK CD56^dim^CD16^**−**^ and a decreased percentage of NK CD56^dim^CD16^+^ cells compared to unvaccinated patients. This difference in CD16 expression by NK cells could be attributed to the fact that this receptor is present on all peripheral blood CD56^dim^ NK cells, but activation of these cells by cross‐linking of CD16 with antibodies results in its loss [27, 28]. Consistently, downregulation of CD16 expression on NK cells, alongside its strong positive association with degranulation, was observed in vivo following intramuscular influenza vaccination. This evidence suggests that CD16 plays a role in the early activation of NK cells following vaccination, as well as in the modulation of NK cell responses and maintenance of immune homeostasis through downregulation [29]. Loss of the CD16 receptor was also observed in recently activated nonclassical monocytes [30]. Hagemann *et al.* have previously demonstrated that antibodies induced by natural SARS‐CoV‐2 infection or vaccination trigger significant NK cell‐mediated ADCC activity [8]. The function of NK cells is regulated by various cytokines, including IFN‐I, which has been shown to be closely associated with the activity of these cells [9, 10]. Consistent with our previous analysis [23], the vaccinated individuals exhibited higher expression of IFN‐I genes than the unvaccinated ones. Notably, none of them had nAbs against IFN‐α or IFN‐ω, regardless of vaccine status. These cytokines have remarkable antiviral activity against SARS‐CoV‐2 [11, 12], although emerging SARS‐CoV‐2 variants have shown progressively increased IFN resistance [31]. Notably, a weak and delayed IFN‐I response is one of the hallmarks of severe forms of COVID‐19 [32]. Furthermore, the number of blood plasmacytoid dendritic cells, which are the main circulating IFN‐α‐producing cell subset, is significantly reduced in severely ill unvaccinated patients hospitalized with SARS‐CoV‐2 [33]. The increased expression of IFN‐I that we observed in vaccinated individuals suggests that they mount a more robust and effective immune response to the virus than unvaccinated individuals. This is also supported by the faster SARS‐CoV‐2 viral clearance observed in the first group after monoclonal antibody therapy. To further clarify this, we performed a correlation analysis between NK cell subset frequencies and IFN‐I mRNA expression levels. We observed a negative correlation between the frequency of NK CD56^dim^CD16^+^ cells and IFN‐I mRNA expression levels. Thus, unvaccinated patients with reduced IFN‐I mRNA levels also exhibited higher frequencies of NK CD56^dim^CD16^+^ cells. This inverse relationship suggests that reduced IFN‐I expression could be associated with impaired NK cell activity. This is consistent with previous findings that IFN‐I plays a crucial role in promoting NK cell activation [9, 10]. In this context, the increased frequency of CD56^dim^CD16^+^ NK cells in unvaccinated individuals may indicate a lower number of activated or functional NK cells, which could be the result of poor IFN‐I signalling. The higher score on the Charlson Comorbidity Index among vaccinated patients could be perceived as a limitation of this study. However, the importance and effectiveness of vaccination are emphasized by the fact that vaccinated patients clear SARS‐CoV‐2 more quickly, despite being frailer. A limitation is that of the 47 patients who had received the SARS‐CoV‐2 vaccine, five had only received the first dose (Supplementary Table S1). However, rather than weakening our findings, this detail actually enhances the robustness of our results, as the impact of the vaccine is already observed after one dose. Another limitation is the lack of quantification of antibodies directed against the Nucleocapsid protein in patient sera, which would have allowed for the exclusion of previous natural SARS‐CoV‐2 infection. However, anti‐nucleocapsid antibodies are not a fully reliable marker of prior infection, due to their variable sensitivity, waning over time, and incomplete seroconversion, particularly in mild or immunocompromised cases [34, 35, 36]. Moreover, given that the patients were enrolled in 2021, that unvaccinated patients did not have anti‐S antibodies in their sera and that asymptomatic SARS‐CoV‐2 infection is unlikely in frail individuals, it is reasonable to presume that none of them had previously been infected, as reported by the patients. In conclusion, our results suggest that SARS‐CoV‐2 infected individuals have different innate immune responses depending on vaccination status. Notably, vaccinated patients demonstrate an early and pronounced IFN‐I response, alongside phenotypic evidence of pre‐activated NK cells in the early stages of infection. Conversely, unvaccinated individuals exhibit an attenuated and delayed activation of these key antiviral pathways, underscoring the immunological priming conferred by prior anti‐S vaccination. Although the underlying mechanisms have not yet been investigated, our findings provide clinically relevant evidence that anti‐S vaccination plays an important role in improving the immune response and reducing the duration of SARS‐CoV‐2 infection.

## Author Contributions

**Giancarlo Ceccarelli:** validation, resources, writing – review and editing. **Valentina Tirelli:** formal analysis, validation, writing – review and editing, methodology. **Eugenio N. Cavallari:** resources. **Mario Picozza:** validation, supervision, writing – review and editing. **Ginevra Bugani:** investigation, visualization, methodology. **Guido Antonelli:** validation, visualization, supervision, funding acquisition. **Luca Maddaloni:** conceptualization, formal analysis, investigation, writing – original draft, writing – review and editing, methodology. **Carolina Scagnolari:** conceptualization, validation, supervision, project administration, writing – review and editing. **Letizia Santinelli:** investigation, visualization. **Claudio M. Mastroianni:** validation, visualization, supervision. **Matteo Fracella:** investigation, visualization. **Gabriella d'Ettorre:** conceptualization, project administration, validation, supervision, funding acquisition, writing – review and editing.

## Funding

This work was supported by EU funding within the NextGeneration EU‐MUR PNRR Extended Partnership initiative on Emerging Infectious Diseases (PE00000007, INF‐ACT).

## Consent

The study was performed following the Institutional Review Board (Department of Public Health and Infectious Diseases, Sapienza, University of Rome) and the Ethics Committee (Comitato Etico Territoriale Lazio Area 1) approval and the signing of informed consent by all study participants (Rif. 5836).

## Conflicts of Interest

The authors declare no conflicts of interest.

## Supporting information


**Table S1:** SARS‐CoV‐2 vaccination status of patients at enrollment.

## Data Availability

The data that support the findings of this study are available from the corresponding author upon reasonable request.

## References

[1] I. Mohammed , A. Nauman , P. Paul , et al., “The Efficacy and Effectiveness of the COVID‐19 Vaccines in Reducing Infection, Severity, Hospitalization, and Mortality: A Systematic Review,” Human Vaccines & Immunotherapeutics 18, no. 1 (2022): 2027160. 35113777 10.1080/21645515.2022.2027160 PMC8862168

[2] K. Rahmani , R. Shavaleh , M. Forouhi , et al., “The Effectiveness of COVID‐19 Vaccines in Reducing the Incidence, Hospitalization, and Mortality From COVID‐19: A Systematic Review and Meta‐Analysis,” Frontiers in Public Health 10 (2022): 873596. 36091533 10.3389/fpubh.2022.873596 PMC9459165

[3] U. Sahin , A. Muik , E. Derhovanessian , et al., “COVID‐19 Vaccine BNT162b1 Elicits Human Antibody and T_H_1 T Cell Responses,” Nature 586, no. 7830 (2020): 594–599. 32998157 10.1038/s41586-020-2814-7

[4] C. Capuano , D. De Federicis , D. Ciuti , et al., “Impact of SARS‐CoV‐2 Vaccination on FcγRIIIA/CD16 Dynamics in Natural Killer Cells: Relevance for Antibody‐Dependent Functions,” Frontiers in Immunology 14 (2023): 1285203. 38045702 10.3389/fimmu.2023.1285203 PMC10693335

[5] Q. Hammer , A. Cuapio , J. Bister , N. K. Björkström , and H. G. Ljunggren , “NK Cells in COVID‐19‐From Disease to Vaccination,” Journal of Leukocyte Biology 114, no. 5 (2023): 507–512. 36976012 10.1093/jleuko/qiad031

[6] V. Gentili , D. Bortolotti , L. Morandi , et al., “Natural Killer Cells in SARS‐CoV‐2‐Vaccinated Subjects With Increased Effector Cytotoxic CD56^dim^ Cells and Memory‐Like CD57^+^NKG2C^+^CD56^dim^ Cells,” Frontiers in Bioscience (Landmark Edition) 28, no. 7 (2023): 156. 37525920 10.31083/j.fbl2807156

[7] D. Mele , S. Ottolini , A. Lombardi , et al., “Long‐Term Dynamics of Natural Killer Cells in Response to SARS‐CoV‐2 Vaccination: Persistently Enhanced Activity Postvaccination,” Journal of Medical Virology 96, no. 4 (2024): e29585. 38566585 10.1002/jmv.29585

[8] K. Hagemann , K. Riecken , J. M. Jung , et al., “Natural Killer Cell‐Mediated ADCC in SARS‐CoV‐2‐Infected Individuals and Vaccine Recipients,” European Journal of Immunology 52, no. 8 (2022): 1297–1307. 35416291 10.1002/eji.202149470 PMC9087393

[9] R. Paolini , G. Bernardini , R. Molfetta , and A. Santoni , “NK Cells and Interferons,” Cytokine & Growth Factor Reviews 26, no. 2 (2015): 113–120. 25443799 10.1016/j.cytogfr.2014.11.003

[10] L. Müller , P. Aigner , and D. Stoiber , “Type I Interferons and Natural Killer Cell Regulation in Cancer,” Frontiers in Immunology 8 (2017): 304. 28408907 10.3389/fimmu.2017.00304 PMC5374157

[11] E. Mantlo , N. Bukreyeva , J. Maruyama , S. Paessler , and C. Huang , “Antiviral Activities of Type I Interferons to SARS‐CoV‐2 Infection,” Antiviral Research 179 (2020): 104811. 32360182 10.1016/j.antiviral.2020.104811 PMC7188648

[12] N. Clementi , R. Ferrarese , E. Criscuolo , et al., “Interferon‐β‐1a Inhibition of Severe Acute Respiratory Syndrome‐Coronavirus 2 In Vitro When Administered After Virus Infection,” Journal of Infectious Diseases 222, no. 5 (2020): 722–725. 32559285 10.1093/infdis/jiaa350 PMC7337790

[13] M. Contoli , A. Papi , L. Tomassetti , et al., “Blood Interferon‐α Levels and Severity, Outcomes, and Inflammatory Profiles in Hospitalized COVID‐19 Patients,” Frontiers in Immunology 12 (2021): 648004. 33767713 10.3389/fimmu.2021.648004 PMC7985458

[14] K. Nagaoka , H. Kawasuji , Y. Murai , et al., “Circulating Type I Interferon Levels in the Early Phase of COVID‐19 Are Associated With the Development of Respiratory Failure,” Frontiers in Immunology 13 (2022): 844304. 35237279 10.3389/fimmu.2022.844304 PMC8882823

[15] C. Scagnolari , A. Pierangeli , F. Frasca , et al., “Differential Induction of Type I and III Interferon Genes in the Upper Respiratory Tract of Patients With Coronavirus Disease 2019 (COVID‐19),” Virus Research 295 (2021): 198283. 33418027 10.1016/j.virusres.2020.198283 PMC7834390

[16] L. Sorrentino , M. Fracella , F. Frasca , et al., “Alterations in the Expression of IFN Lambda, IFN Gamma and Toll‐Like Receptors in Severe COVID‐19 Patients,” Microorganisms 11, no. 3 (2023): 689. 36985262 10.3390/microorganisms11030689 PMC10058642

[17] H. Xia , Z. Cao , X. Xie , et al., “Evasion of Type I Interferon by SARS‐CoV‐2,” Cell Reports 33, no. 1 (2020): 108234. 32979938 10.1016/j.celrep.2020.108234 PMC7501843

[18] P. Bastard , L. B. Rosen , Q. Zhang , et al., “Autoantibodies Against Type I IFNs in Patients With Life‐Threatening COVID‐19,” Science 370, no. 6515 (2020): eabd4585. 32972996 10.1126/science.abd4585 PMC7857397

[19] P. Bastard , Q. Zhang , S. Y. Zhang , E. Jouanguy , and J. L. Casanova , “Type I Interferons and SARS‐CoV‐2: From Cells to Organisms,” Current Opinion in Immunology 74 (2022): 172–182. 35149239 10.1016/j.coi.2022.01.003 PMC8786610

[20] F. Frasca , M. Scordio , L. Santinelli , et al., “Anti‐IFN‐α/−ω Neutralizing Antibodies From COVID‐19 Patients Correlate With Downregulation of IFN Response and Laboratory Biomarkers of Disease Severity,” European Journal of Immunology 52, no. 7 (2022): 1120–1128. 35419822 10.1002/eji.202249824 PMC9087404

[21] M. Scordio , F. Frasca , L. Santinelli , et al., “High Frequency of Neutralizing Antibodies to Type I Interferon in HIV‐1 Patients Hospitalized for COVID‐19,” Clinical Immunology 241 (2022): 109068. 35764258 10.1016/j.clim.2022.109068 PMC9233547

[22] M. Severa , F. Rizzo , A. Sinigaglia , et al., “A Specific Anti‐COVID‐19 BNT162b2 Vaccine‐Induced Early Innate Immune Signature Positively Correlates With the Humoral Protective Response in Healthy and Multiple Sclerosis Vaccine Recipients,” Clinical & Translational Immunology 12, no. 3 (2023): e1434. 36969367 10.1002/cti2.1434 PMC10036198

[23] L. Maddaloni , L. Santinelli , G. Bugani , et al., “Differential Expression of Type I Interferon and Inflammatory Genes in SARS‐CoV‐2‐Infected Patients Treated With Monoclonal Antibodies,” Immunity, Inflammation and Disease 11, no. 10 (2023): e968. 37904704 10.1002/iid3.968 PMC10571496

[24] M. E. Charlson , P. Pompei , K. L. Ales , and C. R. MacKenzie , “A New Method of Classifying Prognostic Comorbidity in Longitudinal Studies: Development and Validation,” Journal of Chronic Diseases 40, no. 5 (1987): 373–383. 3558716 10.1016/0021-9681(87)90171-8

[25] M. Fracella , E. Mancino , R. Nenna , et al., “Age‐Related Transcript Changes in Type I Interferon Signaling in Children and Adolescents With Long COVID,” European Journal of Immunology 54, no. 5 (2024): e2350682. 38522030 10.1002/eji.202350682

[26] A. Ziogas and M. G. Netea , “Trained Immunity‐Related Vaccines: Innate Immune Memory and Heterologous Protection Against Infections,” Trends in Molecular Medicine 28, no. 6 (2022): 497–512. 35466062 10.1016/j.molmed.2022.03.009

[27] R. Romee , B. Foley , T. Lenvik , et al., “NK Cell CD16 Surface Expression and Function Is Regulated by a Disintegrin and Metalloprotease‐17 (ADAM17),” Blood 121, no. 18 (2013): 3599–3608. 23487023 10.1182/blood-2012-04-425397 PMC3643761

[28] B. Grzywacz , N. Kataria , and M. R. Verneris , “CD56(Dim)CD16(+) NK Cells Downregulate CD16 Following Target Cell Induced Activation of Matrix Metalloproteinases,” Leukemia 21, no. 2 (2007): 356–359. 17251901 10.1038/sj.leu.2404499

[29] M. R. Goodier , C. Lusa , S. Sherratt , A. Rodriguez‐Galan , R. Behrens , and E. M. Riley , “Sustained Immune Complex‐Mediated Reduction in CD16 Expression After Vaccination Regulates NK Cell Function,” Frontiers in Immunology 7 (2016): 384. 27725819 10.3389/fimmu.2016.00384 PMC5035824

[30] M. Picozza , L. Battistini , and G. Borsellino , “Mononuclear Phagocytes and Marker Modulation: When CD16 Disappears, CD38 Takes the Stage,” Blood 122, no. 3 (2013): 456–457. 23869076 10.1182/blood-2013-05-500058

[31] L. Laine , M. Skön , E. Väisänen , I. Julkunen , and P. Österlund , “SARS‐CoV‐2 Variants Alpha, Beta, Delta and Omicron Show a Slower Host Cell Interferon Response Compared to an Early Pandemic Variant,” Frontiers in Immunology 13 (2022): 1016108. 36248817 10.3389/fimmu.2022.1016108 PMC9561549

[32] J. Hadjadj , N. Yatim , L. Barnabei , et al., “Impaired Type I Interferon Activity and Inflammatory Responses in Severe COVID‐19 Patients,” Science 369, no. 6504 (2020): 718–724. 32661059 10.1126/science.abc6027 PMC7402632

[33] G. Guerrera , M. Sambucci , E. Timperi , et al., “Identification of an Immunological Signature of Long COVID Syndrome,” Frontiers in Immunology 15 (2025): 1502937. 39845978 10.3389/fimmu.2024.1502937 PMC11750999

[34] A. M. D. Navaratnam , M. Shrotri , V. Nguyen , et al., “Nucleocapsid and Spike Antibody Responses Following Virologically Confirmed SARS‐CoV‐2 Infection: An Observational Analysis in the Virus Watch Community Cohort,” International Journal of Infectious Diseases: IJID: Official Publication of the International Society for Infectious Diseases 123 (2022): 104–111. 35987470 10.1016/j.ijid.2022.07.053 PMC9385348

[35] G. Lippi , B. M. Henry , L. Pighi , S. De Nitto , and G. L. Salvagno , “Are Anti‐SARS‐CoV‐2 S/N IgG/IgM Antibodies Always Predictive of Previous SARS‐CoV‐2 Infection?,” Advances in Laboratory Medicine 4, no. 2 (2023): 175–184. 38075941 10.1515/almed-2023-0008 PMC10701493

[36] E. Grebe , M. Stone , B. R. Spencer , et al., “Detection of Nucleocapsid Antibodies Associated With Primary SARS‐CoV‐2 Infection in Unvaccinated and Vaccinated Blood Donors,” Emerging Infectious Diseases 30, no. 8 (2024): 1621–1630. 38981189 10.3201/eid3008.240659 PMC11286071

