# Supplementary material for: Previous COVID‐19 Vaccination Modulates Type I Interferon and Natural Killer Cell Responses During SARS‐CoV‐2 Infection
Source: J Cell Mol Med. 2026 May 22;30(10):e71190. doi: 10.1111/jcmm.71190 (PMC13240573; doi:10.1111/jcmm.71190)
Supplement: Supplementary file 1 — Table S1: SARS‐CoV‐2 vaccination status of patients at enrollment. [file JCMM-30-e71190-s001.docx]

**Supplementary Table 1** SARS-CoV-2 vaccination status of patients at enrollment

| SARS-COV-2 vaccinated patients | |
| --- | --- |
| **Vaccine type** | **n (%)** |
| • Comirnaty (BNT162b2) | 41/47 (87.2%) |
| 2 doses | 37/41 (90.2%) |
| 1 dose | 4/41 (9.8%) |
| • Spikevax (mRNA-1273) | 2/47 (4.3%) |
| 2 doses | 2/2 (100%) |
| 1 dose | 0/2 (0%) |
| • Vaxzevria (ChAdOx1-S) | 3/47 (6.4%) |
| 2 doses | 2/3 (66.7%) |
| 1 dose | 1/3 (33.3%) |
| • Janssen (Ad26.COV2.S) | 1/47 (2.1%) |

Data are expressed as number (percentage).
